# Polymer Inclusion Membranes Based on Sulfonic Acid Derivatives as Ion Carriers for Selective Separation of Pb(II) Ions

**DOI:** 10.3390/membranes15050146

**Published:** 2025-05-12

**Authors:** Cezary Kozlowski, Iwona Zawierucha

**Affiliations:** Institute of Chemistry, Jan Dlugosz University in Czestochowa, Armii Krajowej 13/15, PL 42200 Czestochowa, Poland; c.kozlowski@ujd.edu.pl

**Keywords:** PIM system, membrane transport, DNNSA, NBSA, Pb(II)

## Abstract

In this paper, polymer inclusion membranes (PIMs) were created using poly(vinyl chloride)-based alkyl sulfonic acid derivatives as ion carriers and dioctyl terephthalate as a plasticizer for the selective separation of Pb(II), Cu(II), and Cd(II) ions from aqueous nitrate solutions. The ion carriers were dinonylnaphthalenesulfonic acid (DNNSA) and nonylbenzenesulfonic acid (NBSA). The influence of the carrier and the plasticizer concentration in the membrane on the transport efficiency was investigated. For the PIM system, 15% wt. of carrier (DNNSA, NBSA), 20% wt. of plasticizer, and 65% wt. of polymer poly(vinyl chloride) PVC were the optimal proportions, with which the process was the most effective. Research on the transport kinetics has shown that the transport of Pb(II) ions through PIMs containing acidic carriers adheres to a first-order kinetics equation, which is characteristic of a facilitated transport mechanism. The activation parameter for these processes suggests that the high performance of these ion carriers is associated with the immobilization of the carrier within the membrane. It was found that PIMs based on DNNSA facilitate the selective separation of Pb(II)/Cu(II) and Pb(II)/Cd(II) mixtures, achieving high separation factors.

## 1. Introduction

Lead(II) must be almost-completely eliminated from wastewater, due to its highly toxic effects. Studies on model solutions have demonstrated that membrane techniques can effectively address this challenge. The contamination of water with lead has gained global attention because of the toxicity and non-biodegradable nature of lead [1]. The World Health Organization has designated a permissible limit of Pb(II) in drinking water of 10 µg/L [2].

The presence of toxic metals in wastewater and post-industrial-discharge waters poses a significant problem for both citizens and scientists in the field of industrial technology. These toxic metals can contaminate the environment through wastewater discharge [3].

Many separation techniques, i.e., extraction, ion exchange, membrane separation, and sorption, have been developed for removal of heavy metals, with varying effectiveness when using traditional extractants [4].

Membrane processes offer an effective alternative for removing heavy metals, as they can achieve high permeate fluxes and high rejection rates while maintaining low energy costs and operating under mild conditions. Additionally, membrane technology allows for continuous separation, and this approach can be easily integrated with other separation methods through hybrid processing.

The increased efficiency attained in the recovery of metals from waste solutions is leading to a shift away from traditional solvent extraction methods, supported liquid membranes (SLMs), and other separation techniques, in favor of PIMs. This change is due to PIMs’ superior stability and minimal carrier loss, and their straightforward design and operation [5,6,7].

A PIM can selectively separate the solutes of interest in a manner similar to SLM but with improved performance. This is because it contains a liquid extractant that functions as a carrier, which is integrated within the membrane’s polymer structure. Unlike SLM, in which the carrier is dissolved in a diluent and held by capillary forces within the relatively large pores of a polymer support, PIM prevents carrier leaching. This characteristic results in PIM having a longer lifespan and greater stability compared to SLM, especially regarding the retention of the membrane’s liquid phase, averting loss of the aqueous phase.

In recent decades, advancements in technology have greatly improved the development of these types of membranes. As a result, PIMs now exhibit not only high efficiency but also selectivity for specific metal ions. This selectivity is particularly important for various industrial applications [8,9].

The modern technique of membrane processes involves the permeation of substances through polymer membranes. The feature most distinguishing PIMs from other membranes is the former’s incorporation of organic phases that contain carriers and plasticizers within the polymer support. This inclusion leads to increased transport efficiency and high selectivity, along with simple applicability and low energy consumption. Additionally, PIMs are often preferred over liquid membranes because PIMs require fewer chemical processes during production and utilize environmentally friendly polymers [10,11].

The investigations often focus on the transport of toxic metal ions, such as lead(II), using a variety of selective ion carriers. Some results from different authors are compiled in Table 1.

Dinonylnaphthalene sulfonic acid (DNNSA) is an acidic carrier commonly used in various industrial applications. The effectiveness of DNNSA, combined with crown ethers used as extractants, was studied by Lamb and Christenson for the removal of strontium ions [21]. They discovered a synergistic effect when both the carriers and the counter-ion were present.

The study by Nazarenko and Lamb [22] found that the membrane transport of Pb(II) is synergistically affected by PIMs containing various crown ethers, in the presence of DNNSA.

Also, a synergetic effect between the calixarenes and DNNSA, the latter used as the counter-ion, was observed in the transport of cesium and strontium ions across PIMs [23].

The DNNSA was also tested as independent cation exchanger for Cs^+^ and Ag^+^ removal in PIM transport [24].

This acidic extractant (DNNSA) has been previously utilized in the solvent extraction of trivalent lanthanides [25], removal of magnesium from phosphoric acid [26], and separation of protactinium(V) from other metal ions [27]. DNNSA has been used as a carrier in PIMs much less frequently than have other commercial extractants. Kozlowski et al. [28,29] applied PIMs containing DNNSA to competitively separate the radioisotopes Co(II), Cs(I), and Sr(II) from aqueous sodium nitrate solutions. Zawierucha et al. [30] investigated the selective transport of Cd(II) and Zn(II) from groundwater through PIMs utilizing DNNSA as an ion carrier.

Plasticizer membranes containing DNNSA were studied to evaluate the impacts of membrane components (plasticizer, carrier, and polymer) on the rate of proton permeation [31]. Because of the high acidity of the carrier, DNNSA exhibits a different affinity for metal ions, compared to commercial acidic extractants like D2EHPA. For example, passive samplers based on PIMs have been developed to determine the time-weighted average concentrations of NH_4_^+^ and Zn(II) in environmental water systems [32,33]. Research has shown that PIMs containing DNNSA exhibit lower permeability for alkali and alkaline-earth ions, compared to those containing D2EHPA.

Ershad et al. [34] demonstrated the selectivity of PIM containing purified DNNSA, 1-tetradecanol, and PVC towards various monovalent and divalent cations in 0.001 M HCl solutions. Selectivity decreases in the following order: Fe(III) > Ca(II) > Pb(II) > Zn(II) > Cu(II) > Ni(II) > Mg(II) > Na(I) ≈ K(I).

Almeida et al. [35] indicated that membranes synthesized using PVC and CTA as polymers exhibit different long-term integrity. It has been observed that PVC-based PIMs are more stable during the transport process.

These results suggest that a more systematic and fundamental study on the selectivity of DNNSA towards common metal ions is necessary in order to explore the potential applications of this ion carrier.

The aqueous solutions used in the steam contained metals such as lead, copper, and cadmium, which are commonly found in the wastewater produced by the metallurgy and mining industries, especially in leachate from flotation dumps.

This work explores the use of DNNSA and NBSA, immobilized in PVC membranes, for lead separation. We studied the kinetic parameters of Pb(II) transport through PIMs that incorporated sulfonic acid derivatives as ion carriers and dioctyl terephthalate as a plasticizer. The competitive transport process for Pb(II), Cu(II), and Cd(II) using DNNSA- or NBSA-PIM membranes demonstrates the potential of a novel separation system for removal of toxic metal ions. The effect of the carrier and the plasticizer concentration on the transport efficiency was also examined.

## 2. Materials and Methods

### 2.1. Chemicals

Reagent grade lead(II), cadmium(II), and copper(II) nitrates, as well as the nitric acid used for the preparation of solutions, were purchased from POCh (Gliwice, Poland). The organic reagents, namely, the polymer PVC, the plasticizer—dioctyl terephthalate (purity > 97%)—and tetrahydrofuran (THF), were purchased from Sigma Aldrich Fluka Chemical Co. (Louis and Burlington, MA, USA); they were used as received. The DNNSA was used as an ion carrier in the membranes as received. It was obtained from the supplier as a 50 wt% solution in n-heptane (Sigma Aldrich, Hamburg, Germany). NBSA (purity > 99%) was purchased from EvitaChem, Pasadena, CA, USA. Additional solvents, such as acetone and dichloromethane, along with other chemicals used in the research, were sourced from Merck KGaA, Poland, while ensuring that they were of the highest purity available.

### 2.2. Solvent Extraction Procedure

An aqueous solution of lead(II) salt cation (10 cm^3^ of 1.0 × 10⁻^3^ M in HNO_3_) was combined with 10 cm^3^ of 0.15 M extractants, either DNNSA or NBSA, in dioctyl terephthalate. The mixture was shaken vigorously in a stoppered glass tube using a mechanical thermostat–shaker (IKA KS 3000i, IKA, Guangzhou, China) at a speed of 300 rpm for 60 min, and then allowed to stand for an additional 60 min. Each lead ion solvent extraction was performed three times. The concentration of Pb(II) in the liquid–liquid extraction was measured using an atomic absorption spectrophotometer (FAAS). Determinations of Pb(II) were carried out with a Solar 939 (Unicam, Munich, Germany) spectrometer with pneumatic nebulization and mono-element lamps, with a hollow cathode made by Unicam for flame atomic-absorption spectrometric analysis. Hollow cathode lamps were used throughout the study and D2-background correction. In the FAAS determinations, the lead peak was located in the area of 283.3 nm wavelength.

### 2.3. Membrane Preparation

The organic solutions containing support—PVC, the ion carriers (DNNSA or NBSA), and the plasticizer (dioctyl terephthalate) in THF were prepared. To synthesize the PIMs, the following solutions were used: 50 mg PVC in THF, 10% dioctyl terephthalate *v*/*v* in THF, and 0.10 M solution of the ion carriers in THF. The specified volumes of the PVC solution, plasticizer, and carrier were mixed. The solutions were stirred continuously for at least 2 h using a magnetic stirrer, resulting in solutions with a homogeneous cast. A membrane mold consisting of a 6.0 cm glass ring bonded to a glass plate using PVC-THF glue was filled with a part of this solution. The mold was covered with a small glass plate that permitted aeration while preventing cross-contamination. It was left at room temperature for over 24 h to allow the slow evaporation of the THF, resulting in the formation of a PIM as a thin film. Once the solvent had completely evaporated, the membrane was carefully removed from the glass plate and conditioned in 100 mL of distilled water for 24 h before use. Subsequently, measurements were taken to assess transport properties. Three identical membranes for each of experiments were synthesized and used for transport measurements.

### 2.4. Kinetic Parameters Describing the Transport Process

Metal ions are transported across the membrane from the source phase to the receiving phase by the counter-transport mechanism, whereas hydrogen ions are transported in the reverse way. The metal ion and the deprotonated form of the carrier combine to form a complex compound called MLn at the source phase/membrane interface. Following the complex compound’s diffusion at the membrane/receiving phase interface, the metal ions are back-extracted into the receiving phase while the hydrogen ions in this phase are simultaneously taken in.

The difference in the partition coefficient values, which are created by keeping a suitable pH gradient between the source and receiving phases, is what promotes the counter-transport process. The following are the main parameters that describe how ions are transported via the polymer membrane: the initial flux of metal ions—J_0_—and the S_M1/M2_ selectivity coefficients and the percentage of metal ion separation—RF [36,37,38].

The initial metal ions flux J_0_ is defined by Equation (1):

(1) $$J_{0}=Pc_{0},$$

(2) $$P=(\frac{V}{A})k,$$

where P is the permeability coefficient and c_0_ is the initial metal ions concentration in the source phase (mol/dm^3^).

The reaction rate constant k (s^−1^) is defined by the different values determining the order of the reaction. The first-order reaction occurring in the membrane is described in Equation (3).

(3) $$\ln\frac{c}{c_{0}}=-\mathrm{kt}$$

The transport of metal ions across the PIM is characterized by the selectivity coefficients S_M1_/_M2_, quantified by Equation (4) (where J_0,M1_ and J_0,M2_ are the initial flux for the M1 and M2 metal ions, respectively).

(4) $$S_{M1/M2}=\frac{J_{0,M1}}{J_{0,M2}}.$$

Another equally important parameter is the recovery factor RF, showing the efficient removal of metal ions from the source phase to the receiving phase (Equation (5)):

(5) $$\mathrm{RF}=\frac{c_{0}-c}{c_{0}}100\%,$$

where c_0_—initial concentration of metal ions in the source phase (mol/dm^3^), c—metal ion concentration after the period of time (mol/dm^3^).

### 2.5. Transport Studies

**Figure 1:**
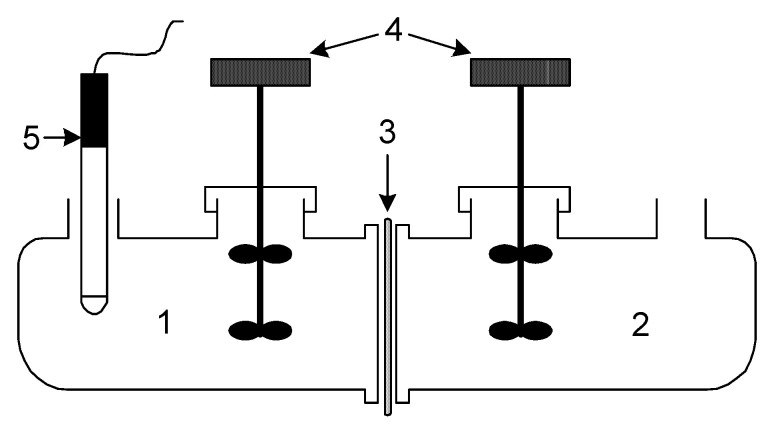
Diagram of the transport experiments across the PIM: 1—source phase; 2—receiving phase; 3—membrane; 4—mechanical stirrer; 5—pH electrode with pH meter.

Transport tests were conducted in a permeation cell with two equal cylindrical compartments (half-cell capacity 50 cm^3^), as shown in Figure 1. The source phase was an aqueous nitrate solution containing 1.0·10^−3^ M Pb(II). A solution of 2.0 M HNO_3_ served as an acceptor phase. In the corresponding cell compartments that the prepared PIM divided, equal volumes (50 cm^3^) of the donor and acceptor phases were added. The membrane’s diameter was 6.0 cm.

The source phase acidity was controlled by pH meter (multifunctional pHmeter, CX-731 Elmetron, with integrated pH electrode, ERH-136, Hydromet, Stróże, Poland). Elemental analyses were conducted using AAS (Solar 939, Unicam, Munich, Germany). The average CTA membrane thickness at 2.0 cm^3^ plasticizer per 1.0 g PVC was 26 μm (measured by digital ultrameter of the A2002M type from Inco-Veritas with a 1.0 μm standard deviation over four readings). The competitive transport experiments were carried out using PIMs containing DNNSA or NBSA for the following nitrate solutions of cations: 1.0·10^−3^ M Pb^2+^, Cd^2+^ and Cu^2+^ (pH = 3.0).

### 2.6. Contact Angle Measurements

The water contact angles (θ) of the PIMs with the DNNSA or NBSA were measured using an OCA 15 Pro goniometer (DataPhysics, Filderstadt, Germany), with the analysis conducted through SCA 20 software, utilizing the sessile drop technique. Each water droplet had a volume of 2.4 μL, and at least four spots on each PIM were analyzed to calculate an average contact angle. The measurements were taken at a temperature of 25 °C.

### 2.7. Atomic Force Microscope Images of PIMs

A surface characterization of the PIMs was performed using an atomic force microscope (AFM) (Digital Instruments Vecco Metrology Group, Santa Barbara, CA, USA) according to the procedure described by Zawierucha et al. [39].

## 3. Results and Discussion

### 3.1. Solvent Extraction of Pb(II) by Sulfonic Derivative Acids

**Figure 2:**
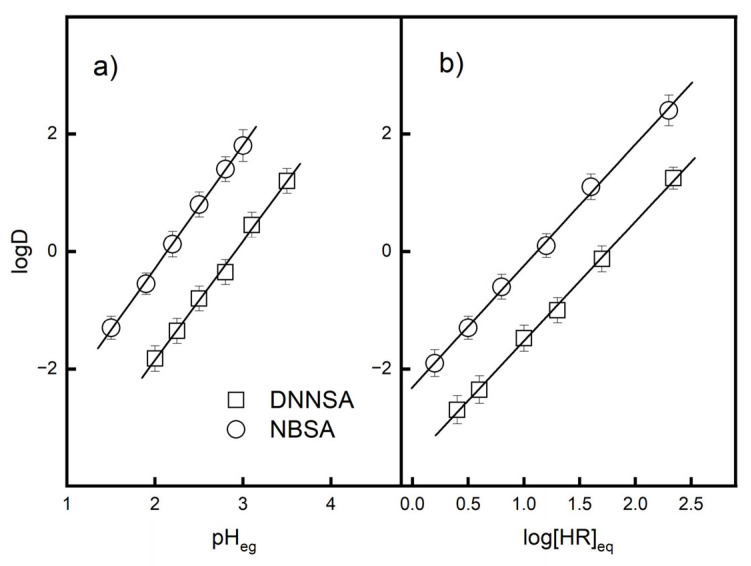
Relationship determined from extraction equilibria: (**a**) effect of the equilibrium pH on the distribution ratio of Pb(II) using DNNSA or NBSA. [Extractants] = 0.15 M, [Pb] = 0.001 M; (**b**) effects of the concentrations of DNNSA or NBSA on the distribution ratio of Pb(II), pH_eq_ 2.0, [Pb(II)] = 0.001 M. The bar graphs indicate the standard deviation values for three measurements.

The stoichiometry of the equilibrium reaction equation for solvent extraction of Pb(II) with DNNSA or NBSA in dioctyl terephthalate was investigated using slope analysis. Figure 2a shows the effect of equilibrium pH on the extraction of Pb(II) using DNNSA or NBSA. The logarithm of the distribution ratio increased with increasing equilibrium pH. The slope of the relationship between logD and the equilibrium pH was 2 for both extractants, which indicates that two hydrogen ions were released from the extractant into the aqueous phase.

Therefore, the extraction data as a function of the extractant concentration were analyzed using plots of logD versus the logarithm of the equilibrium concentrations of the DNNSA and NBSA. Figure 2b shows the effects of the concentrations of DNNSA and NBSA on the distribution ratio of Pb(II). The slope of the relationship between logD and the logarithm of the equilibrium concentrations for these acid extractants is 2, which indicates that two molecules take part in the extraction of Pb(II). The stoichiometry for DNNSA agrees with those reported in previous papers [29,34].

Based on the results of slope analysis, the extraction reaction for Pb(II) using DNNSA and monomer NBSA can be expressed as Equation (6):

(6) $${\mathrm{Pb}}^{2+}+2\mathrm{HR}\vec{\leftarrow }\;\mathrm{Pb}(R{)}_{2}+2H^{+}$$

The extraction equilibrium constant K_ex_ is given by Equation (7):

(7) $$K_{\mathrm{ex}}=\frac{[\mathrm{Pb}(R{)}_{2}][H^{+}{]}^{2}}{\left[{\mathrm{Pb}}^{2+}\right][\mathrm{HR}{]}^{2}}$$

and the distribution ratio of Pb(II) between the organic and aqueous phases is defined as

(8) $$D=\frac{[{\mathrm{Pb}}^{2+}{]}_{\mathrm{aq},\mathrm{eq}}}{[{\mathrm{Pb}}^{2+}{]}_{\mathrm{org},\mathrm{eq}}}=\frac{[\mathrm{Pb}(R{)}_{2}]}{\left[{\mathrm{Pb}}^{2+}\right]}$$

By combining Equations (7) and (8), Equation (9) in its logarithmic form is obtained:

(9) $$\mathrm{logD}=2\mathrm{pH}+2\log\left[\mathrm{HR}\right]+\log K_{\mathrm{ex}}$$

**Figure 3:**
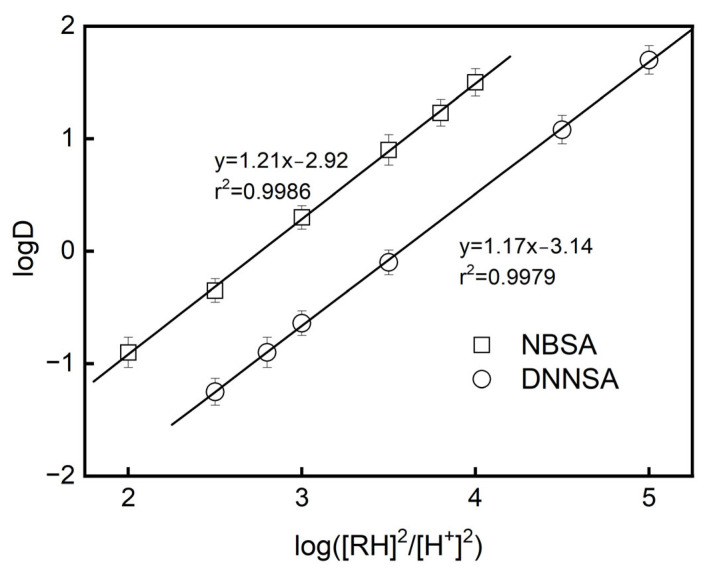
Effect of equilibrium pH and the concentrations of extractants on the distribution ratio of Pb(II). The bar graph indicates the standard deviation values for three measurements.

The experimental logD data for the extraction of Pb(II) using DNNSA or NBSA are plotted in Figure 3.

The extraction equilibrium constants Kex of Pb(II) were evaluated based on the intercept of the straight line with the ordinate of Figure 3, resulting in logK_ex_ = 2.92 for NBSA and logK_ex_ = 3.14 for DNNSA in dioctyl terephthalate, respectively.

### 3.2. Kinetics in the Transport of Pb(II) Across PIM

**Figure 4:**
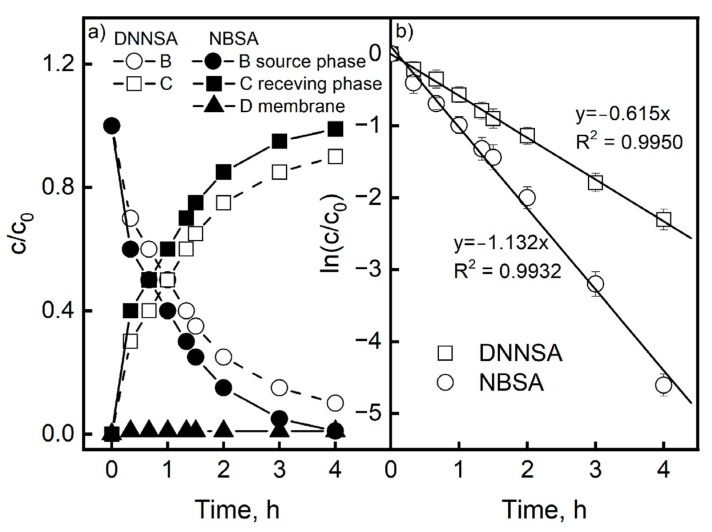
The profile of Pb(II) concentrations in the source, membrane, and receiving phases during the transport process across the PIM with DNNSA or NBSA (**a**) and kinetics curves of Pb(II) transport from the source phase (**b**). Source phase: 1.0·10^−3^ M Pb(NO_3_)_2_ (pH = 3.0); membrane: 2.0 cm^3^ plasticizer/1.0 g PVC; 10% wt. carrier; receiving phase: 2.0 M HNO_3_. The bar graph shows the standard deviation values from three measurements.

For the transport of Pb(II) ions via PIMs with DNNSA or NBSA, the rates of change of the Pb(II) ion concentrations in the aqueous source, aqueous receiving phase, and membrane were examined, yielding a concentration profile of the metal as a function of time (Figure 4). There was no metal buildup in the membrane case, as shown in Figure 4a, confirming that the rates of Pb(II) transport to and from the membrane were rapid and comparable. Figure 4b shows the curves of c/c_0_ as a function of time. The curves have an exponential character, which confirms the kinetic model of the first-order transport of metal ions proposed by Danesi et al. [40], which is analogous to that used for liquid membranes. The ln(c/c_0_) = f(t) dependencies represent the slopes of the straight lines, and for each membrane used, the average constants of the transport rates (k) were estimated. Based on the obtained values, the initial fluxes of Pb(II) transport (J_0_) were obtained. The kinetic curves of these processes are shown in Figure 4b. The determination coefficients R^2^ of the linear dependencies ln(c/c_0_) as a function of time for the DNNSA and NBSA were 0.9950 and 0.9932, respectively. Therefore, these dependencies were determined at a high level of statistical significance. Based on the Pb(II) transport measurement results, the average values of the initial fluxes and their standard deviations were determined; they were 18.85 ± 0.05 µmol/m^2^s for DNNSA and 22.17 ± 0.08 µmol/m^2^s for NBSA. The reported values represent the averages of three replications, and have a standard deviation of no more than 2%.

The diffusional flux at the membrane phase J_m_ for DNNS and NBSA complex Pb(R)_2_ can be written as

(10) $$J_{m}=\Delta_{m}^{-1}([\mathrm{Pb}(R{)}_{2}{]}_{m/s}$$

The local equilibrium at the interface is achieved, and the concentrations at the interface are described by Equation (6).

Thus, at the steady state, J_aq,source_ = J_o_ = J, the flux for DNNSA and NBSA can be obtained as

(11) $$J=\frac{{\left[C\right]}_{\mathrm{org}}(K_{\mathrm{ex}}/{[H}^{+}])}{\Delta_{m}+\Delta_{\mathrm{org}}[C{]}_{\mathrm{org}}(K_{\mathrm{ex}}/[H^{+}])}[M^{n+}]$$

where [C]_0_ is the initial concentration of DNNSA or NBSA, and the total free carrier concentration of [C]_org_ is

(12) $$[C{]}_{\mathrm{org}}=[C{]}_{0}-[\mathrm{MC}{]}_{\mathrm{org}}$$

Diffusion is limited by processes occurring in the organic phase of membrane, which is immobilized in the polymer matrix; the equation for transport flux is as follows [29,30]:

(13) $$J=\frac{[C{]}_{0}}{\Delta_{0}}=\frac{d[M^{n+}]}{\mathrm{dt}}\frac{V}{A}$$

While the carrier concentration in the PIM was 0.25 M DNNSA and the complex concentration equal to only 10^−5^ M, it could be assumed that [C] in Equation (11) is practically equal to [C]_0_. Integrating Equation (13) in relation to the metal concentration for the time from t = 0 to t, the correlation is established as follows:

(14) $$[M^{n+}{]}_{0}-[M^{n+}{]}_{t}=(A\left[C{]}_{0}/V\Delta_{0}\right)t$$

**Figure 5:**
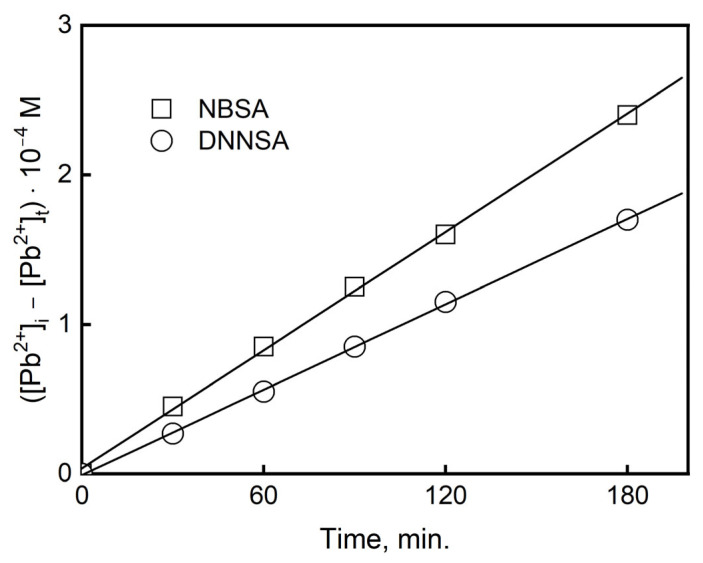
Relation of [Pb(II)]_i_ − [Pb(II)]_t_ vs. time for metal transport across a PIM with 10% wt. DNNSA or NBSA. The conditions of the experiment were as follows: the source phase, 1.0·10^−3^ M Pb(II) solution at pH 3.0; the receiving phase, 2.0 M HNO_3_ solution; and the PIM, 2.0 cm^3^ dioctyl terephthalate/1.0 g PVC.

The regression coefficient is calculated on the basis of the relationship [M^n+^]_0_ − [M^n+^]_t_ vs. the time of the process (t)—Figure 5. The regression coefficient (A[C]_0_/VΔ_o_) is obtained from Equation (14). The diffusion coefficient of the metal complex in the membrane (D_o_) can be calculated when the thickness PIM (d_o_) and the value of the diffusion resistance (Δ_o_) are known, as follows:

(15) $$D_{o}=\frac{d_{o}}{\Delta_{o}}$$

We determined the diffusion coefficients of the metal complexes for the DNNSA and NBSA in the membrane by knowing the diffusive resistance and the membrane thickness (d_o_ = 28 µm).

The values of the diffusion coefficients for the PIMs determined in this study were equal for DNNSA D_o_ = 4.58 10^−8^ cm^2^/s and NBSA D_o_ = 1.95 10^−8^ cm^2^/s, and show that the limiting step of the process is the permeation of the lead(II) complex through the membrane barrier.

The degree to which the carriers—the components of “optimal” PIM—leached from the membrane (after eight cycles of transport) was used to assess the stability of the material. With a membrane mass loss of 4 ± 1% (*n* = 3), the components of the membrane exhibited excellent resistance to migration from the base polymer. Moreover, the eight repeated cycles of Pb(II) extraction were conducted using the same membranes (with DNNSA or NBSA) to evaluate their reusability. These measurements were performed over 4 h by refreshing the source and receiving phases at the end of each cycle. The results indicate that the efficiency of the PIMs is reproducible. The RF values were above 95–97% in the first four cycles of the PIMs (each cycle lasting 4 h), but then slightly decreased to 90%.

### 3.3. Effect of Temperature

The initial flow can be related to the temperature by use of the Arrhenius law, as follows:

(16) $$J\left(T\right)=\mathrm{Aexp}(\frac{{-E}_{a}}{\mathrm{RT}})$$

where R denotes the perfect gas constant, A denotes the pre-exponential factor, and E_a_ denotes the activation energy for the formation of the transition state related to the kinetically determining step, which is the diffusion of the metal complex through the membrane [41,42,43].

After linearization, Equation (14) can be expressed as

(17) $$\ln J_{0}=-\frac{E_{a}}{R}\left(\frac{1}{T}\right)+\mathrm{lnA}$$

From the slope of the curve lnJ_0_ = f(1/T), Ea can be computed. According to the activated-complex theory, the following equation links Ea to the enthalpy of activation ΔH:

(18) $$\Delta H=E_{a}-2500\;\mathrm{at}\;25\;{}^{\circ}C$$

While the entropy ΔS is linked to the pre-exponential factor by the following equation:

(19) $$\Delta S=R\left(\mathrm{lnA}-30.46\right)\;\mathrm{at}\;25\;{}^{\circ}C$$

**Figure 6:**
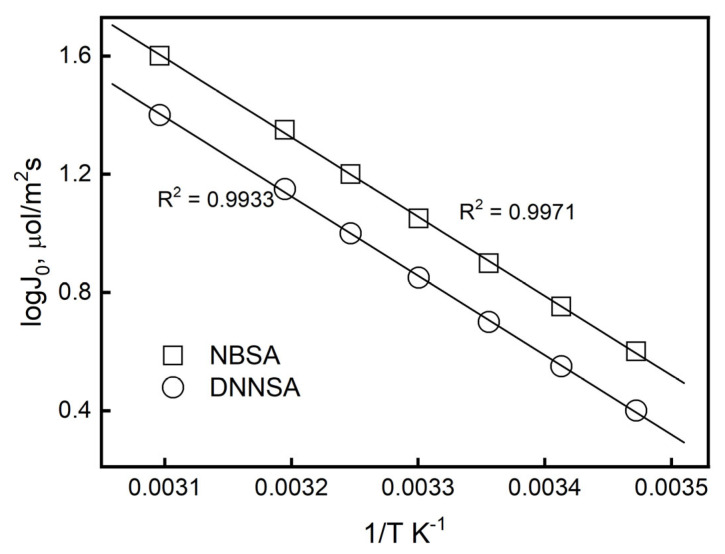
Arrhenius plot of Pb(II) transport through the PIM containing DNSSA or NBSA.

One parameter used to classify step-controlled mechanisms is transport activation energy (Ea). When Ea < 20 kJ mol^−1^, the process was regulated by diffusion; for Ea > 42 kJ mol^−1^, the chemical reaction was used. Both diffusion and chemical reaction controlled the transport Ea in the 20 ÷ 42 kJ mol^−1^ range [44,45]. The transport of Pb(II) by the PIM at temperatures between 288 K and 323 K was investigated in this work. Figure 6 illustrates how the J_0_ rises as the temperature rises. The Arrhenius law, Equation (15), was used to determine the activation energy, which was 28.67 kJ mol^−1^ for DNSSA and 33.71 kJ mol^−1^ for NBSA. This suggests that Pb(II) movement across the PIM with the ion carriers under investigation is controlled by both chemical reaction and diffusion.

As the temperature rose, the metal ion transport rate through the PIM increased as well. The solute’s molecular motion and its polymer chain’s thermal motion increased, the transport system’s viscosity decreased, and the rate of chemical reactions accelerated as a result of the temperature rise [46]. The high Ea in this investigation also suggested that the transport process was impacted by the chemical complexation reaction between the carrier and Pb(II). Table 2 displays the activation parameters (activation energy Ea, activation enthalpy ΔH, and activation entropy ΔS) associated with the enhanced transport of Pb(II) ions through the membranes with DNNSA or NBSA, which were computed from the slopes and intercepts of these lines.

In the complexation/decomplexation reaction between the metal ion and the carrier, the activation entropies that corresponded to the transition state had negative values. These findings demonstrated that a closer approach between the metal ions and the ion carrier in the transition state resulted in a gain of order, suggesting an early transition state. The values obtained for this parameter (ΔS) for the membranes under study were extremely close, indicating that the Pb(II) ions’ reaction with the ion carrier molecules during the ions’ passage through the membranes took place in accordance with the same sites of interaction determined between the lead ion and the DNNSA and NBSA acids. Conversely, low ΔH values suggested low activation energy for these transition states in both membranes, resulting in quick responses at the membrane/aqueous interface. Therefore, the structural aspect had a significantly greater impact than the energy aspect on the directed process of enhanced transport of Pb(II) ions across these plasticizer membranes.

### 3.4. Determination of Optimum Carrier and Plasticizer Contents in the Membrane

**Figure 7:**
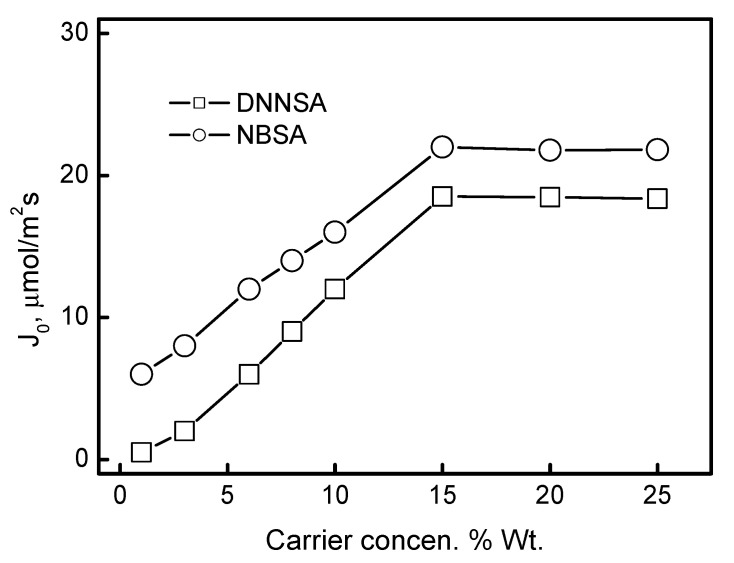
The lead(II) initial fluxes obtained after 4 h of transport across PIM with DNNSA or NBSA at different concentrations. The source phase: 50 cm^3^ of model aqueous solution; the receiving phase: 50 cm^3^ of 2.0 M HNO_3_ solution; PIM: 2.0 cm^3^ dioctyl terephthalate/1.0 g PVC at different carrier concentrations.

The influence of the concentration of the sulfonic acid derivative immobilized in PIM on the permeation of Pb(II) ions was analyzed. Similarly to the previous studies, the source phase was a solution of 1.0∙10^−3^ M Pb(II) at pH = 3.0, and the receiving phase was a 2.0 M HNO_3_ solution. Membranes with constant contents of PVC (50 mg) and plasticizer (2 cm^3^ dioctyl terephthalate/1.0 g PVC) were prepared, while the concentration of the carrier in the membrane varied from 1% to 25% wt. The studies were carried out for 4 h. Membranes without the carrier did not transport metal ions, which indicates that its concentration in the membrane affects the facilitated transport of metal ions by PIM. The dependence of the Pb(II) ion transport flux on the carrier concentration in PIM is shown in Figure 7. A carrier concentration in the membrane above 15% causes the polymer membrane to be saturated with the carrier. The initial fluxes of transport for this concentration were maximal and then became practically constant. For the carriers DNNSA and NBSA the maximal values of the fluxes were 18.85 µmol/m^2^s and 22.70 µmol/m^2^s, respectively.

**Figure 8:**
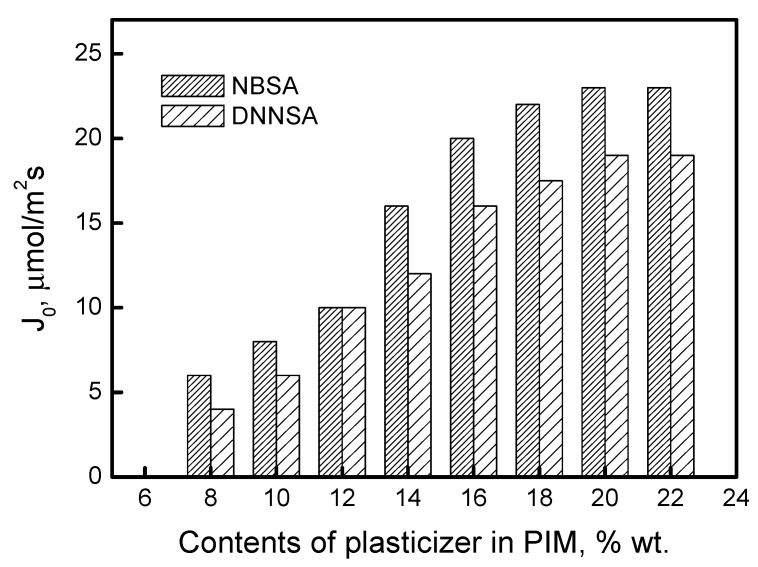
The effect of plasticizer concentration on Pb(II) transport across PIMs with DNNSA or NBSA.

Plasticizers are well-known chemical compounds applied in polymer manufacturing to ensure flexibility and eliminate the effects of brittleness and cracking. In this study, dioctyl terephthalate as a plasticizer was tested in order to verify its effect on the lead(II) transport through PIMs. Figure 8 shows the changes in the Pb(II) transport fluxes, depending on plasticizer concentration, in the PIMs with DNNSA or NBSA.

The permeability relations shown in Figure 8 demonstrate a plasticization effect. As the amount of plasticizer increases, there is a corresponding rise in permeability. Based on the data presented in Figure 8, PIMs with DNNSA or NBSA and containing 20% wt. of the plasticizer were selected and prepared for further investigation.

In this context, the plasticizer enhances the membrane’s ability to facilitate the mobility of the extractant. Determining the concentration of plasticizer in the membrane is very important. According to several reports in the literature, increasing the plasticizer concentration can positively affect the metal ion transfer rate, but only within a specific range of concentrations. Gherrou et al. [47] showed that the initial flux of Cu(II) ion transfer through a CTA membrane containing dibenzo18-crown-6 ether increases with increasing plasticizer (2-Nitrophenyl pentyl ether) concentrations in the membrane only in the range of 0 to 10 mg/cm^2^ of membrane surface. In turn, Kozlowski et al. [48] have shown that, for transport of chromium(VI), the optimal o-nitrophenyl pentyl ether concentrations when used as plasticizer with tri-n-octylamine and 4-(1-n-tridecyl)pyridine N-oxide are 0.8 and 4.0 cm^3^ o-nitrophenyl pentyl ether/1 g CTA, respectively.

Both excessive and insufficient amounts of plasticizer in the PIM are undesirable. The anti-softening effect of a low plasticizer content in the PIM makes the membrane brittle. However, if the membrane contains an excessive amount of plasticizer, the additional plasticizer may flow into the aqueous phase and produce a film that facilitates the movement of metal ions at the membrane–aqueous phase interface.

### 3.5. Competitive Transport of Pb(II), Cu(II), and Cd(II) by PIMs with DNNSA or NBSA

The competitive transport of Pb(II), Cu(II), and Cd(II) from nitrate solutions of metal ions, each at a 1.0·10^−3^ M concentration, across PIMs with DNNSA or NBSA was also investigated. The recovery factors, kinetic parameters, and selectivity order are summarized in Table 3. The transport rates of Pb(II), Cu(II), and Cd(II) across the studied membrane decreased in the following order: Pb > Cu > Cd. Higher selectivity of Pb(II) from Cu and Cd was observed using the PIM with DNNSA; then, they were S_Pb/Cu_ = 111 and S_Pb/Cd_ = 222, respectively. Slightly lower selectivity was observed for PIM with NBSA, while the process efficiency was higher.

The DNNSA-based PIM extraction mechanism for the studied cations is assumed to involve the dissociation of DNNSA at the PIM surface/solution interface, which is facilitated by a decrease in the pH of the solution. The extraction efficiency is also strongly affected by the metal cation’s ability to form an ion-pair with the conjugated base of DNNSA. This can be assessed based on the Hard and Soft Acids and Bases Theory, which suggests that more-electron-dense and harder-to-polarize metal cations (e.g., Pb) can form ion-pairs with the conjugated base of DNNSA more readily, while less-electron-dense and more-easily polarized metal cations (such as Cu and Cd) are more difficult to extract.

The values of the separation coefficients obtained using PIM with DNNSA and NBSA are much higher than those described in the literature for membrane systems with D2EHPA and Cyanex 301 [11,12]; the values of the latter are lower than 50. On the other hand, PIMs containing macrocyclic compounds [17,18] (calixresorcinarene, cyclodextrin polymer) are selective towards Pb(II) (selectivity factor lower than 100), although the metal transport rate is an order of magnitude lower.

### 3.6. Characterization of Membrane Surface

**Figure 9:**
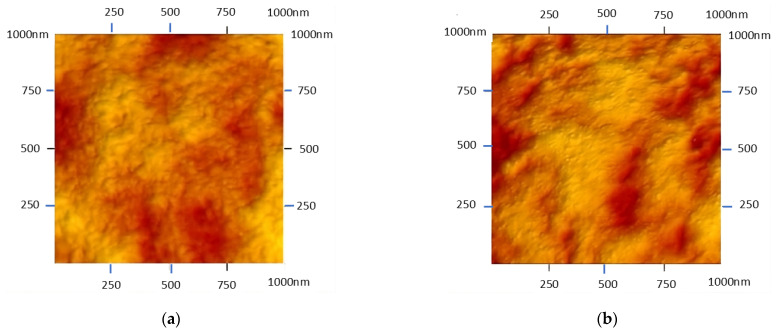
AFM pictures of the PIMs based on PVC with (**a**) DNSSA and (**b**) NBSA as ion carriers.

One of the important aspects characterizing the transport through PIMs is the microstructure of the membrane surface, which can be determined using AFM. Figure 9 shows 2D photos of a membrane with a surface of 1 µm × 1 µm, containing, in addition to the PVC matrix, a plasticizer and ion carriers (DNNSA or NBSA). The pictures show organic phase inclusions in the form of large pores with a diameter of about 0.05 µm.

From the presented topography of the membrane synthesized using NBSA as a carrier and dioctyl terephthalate as a plasticizer, it can be seen that the pore size with the included organic phase is slightly larger compared to the membrane with DNNSA. The estimated pore sizes of the PIM membrane for DNNSA and NBSA, as well as plasticizer, are 0.065 µm and 0.074 µm. In the case of the PIM with the investigated sulfonic acids, the resulting pores have the shape of elongated drops, and the porosity is about 55%. The degrees of folding of the membranes containing the carrier, DNNSA or NBSA, as determined by the parameter (R_a_), were 5.27 nm and 6.23 nm, respectively. This can also indicate a slight influence of the carrier on the formation of membrane pores through the interactions of the matrix with the carrier structure.

Wettability reflects the hydrophilic and hydrophobic properties of the membrane surface; these properties are associated with both the matrix and the organic phase immobilized on it, and containing a hydrophobic carrier. The determined values of the wetting angles of the PIMs containing DNNSA and NBSA carriers were 44.27° and 48.75°, respectively, which proves the hydrophilic character of the plasticized PVC membranes. In this case, wetting angles above 90° were not observed, so they are not hydrophobic, and the differences in the values of the wetting angles are caused by the structure of the carrier molecule, as well as the porosity on the membrane’s surface. The structure of the DNNSA molecule containing two alkyl substituents causes a slight increase in the wetting angle due to an increase in the hydrophobicity of the carrier molecule. The determined contact angles of the PIM membranes containing ionic liquids are similar to those obtained by Baczynska et al. [49], and range from 35° to 37°.

## 4. Conclusions

In this study, transport of Pb(II) through PIMs was performed using DNNSA and NBSA derivatives as ion carriers. It was found that sulfonic acid derivatives efficiently extract lead ions. For the PIM system, it was found that a carrier concentration of 15% wt. of DNNSA or NBSA was the most effective. Under the optimal conditions for the PVC-based PIM (i.e., source phase: 1.0·10^−3^ M Pb(NO_3_)_2_ of pH = 3.0; PIMs: 65% PVC, 20% plasticizer, and 15% carrier (wt.); receiving phase: 2.0 M HNO_3_), the transport efficiency for Pb(II) ions was 96–99% after 4 h. The transport of Pb(II) through the PIMs using DNNSA or NBSA is facilitated and follows first-order kinetics. Thermodynamic experiments show that the transport of Pb(II) across the PIM is governed by both diffusion within the membrane and a chemical reaction. Higher selectivity of Pb(II) from Cu(II) and Cd(II) was observed using transport across the PIM with DNNSA and NBSA.

Optimized PIMs selectively extract lead ions from synthetic solutions in the order of Pb^2+^ > Cu^2+^ > Cd^2+^, confirming the potential of a novel separation system for wastewater treatment applications.

The increasing demand for effective methods to remove toxic metal ions from polluted streams has led to a heightened interest in techniques that utilize PIM systems. However, additional research is needed to address challenges related to the long-term integrity of these membranes and their potential for repeated use; these challenges include the development of affordable and efficient membrane regeneration methods. Another important factor when considering the large-scale application of PIMs is the creation of environmentally friendly and cost-effective methods for managing used membranes. Addressing the issues related to the stability and efficiency of PIM systems will undoubtedly promote their broader adoption in the future for removing various pollutants from aquatic environments.

## Figures and Tables

**Table 1:** Comparison of lead(II) selectivity in PIM systems.

Carriers	Selectivity Order	Selectivity Factor Pb/M^n+^	Refs.
Cyanex 301	Pb(II) > Cd(II) > Zn(II)	40	[11]
D2EHPA	Pb(II) > Cd(II) ≈ Zn(II)	60	[12]
Kelex 100	Pb(II) > Cd(II)	50	[13]
PNP-16C6	Pb(II) > Zn(II) > Cd(II)	80	[14]
Calix[4]arene	Hg(II) > Pb(II) > Cd(II)	70	[15]
Benzoic acid ester	Pb(II) ≫ K(I) ≈ Ca(II) ≈ Na(I)	>100	[16]
β-CD deriv	Pb(II) > Zn(II) ≈ Cu(II)	100	[17]
Calixresorcin[4]arene	Pb(II) > Cd(II), Zn(II)	40	[18]
Calixresorcin[4]arene	Pb(II) > Cd(II) > Zn(II)	75	[19]
D2EHPA/Aliquat 336	Zn(II) > Cd(II) > Pb(II)	-	[20]

**Table 2:** Activation parameters related to the facilitated transport of Pb(II) ions through the PIMs.

Parameters	PIM_DNNSA_	PIM_NBSA_
Activation energy Ea, kJmol^−1^	28.67	33.71
Activation entropy ΔS, Jmol^−1^K^−1^	−280.25	−303.81
Enthalpy of activation ΔH, kJmol^−1^K^−1^	12.52	16.09

**Table 3:** The initial fluxes, recovery factor after 4 h and selectivity order for the competitive transport of Pb(II), Cu(II), and Cd(II) ions across PIMs doped with DNNSA or DNSA. Membrane, 20% wt; plasticizer, 15% wt.; carrier 65% wt. PVC. Source phase: [M^2+^] = 0.001 mol/dm^3^ for each metal ion; receiving phase: 2.0 mol/dm^3^ HNO_3_.

Carriers	Metal Ions	RF, %	J_0_, µmol/m^2^s	Selective Order Selective Coefficients
DNNSA	Pb	96.3	20.1	Pb > Cu > Cd
	Cu	10.6	0.18	S_Pb/Cu_ = 111
	Cd	1.7	0.09	S_Pb/Cd_ = 222
NBSA	Pb	98.5	22.2	Pb > Cu > Cd
	Cu	4.3	0.32	S_Pb/Cu_ = 68
	Cd	0.2	0.12	S_Pb/Cd_ = 183

## Data Availability

The original contributions presented in the study are included in the article; further inquiries can be directed to the corresponding author.
